# Vitamin D Pathway Activation Reduces Cardiomyocyte DNA Damage and Improves Cardiac Contractility in Preclinical Models

**DOI:** 10.1016/j.jacadv.2026.103199

**Published:** 2026-09-09

**Authors:** Masamichi Ito, Manami Katoh, Masayuki Kubota, Masataka Yamasaki, Yanfei Zhang, Jin Komuro, Ayako Tsukihashi, Shintaro Yamada, Toshiyuki Ko, Mikako Katagiri, Ryo Abe, Shunsuke Inoue, Takashi Hiruma, Daigo Nishijo, Zhehao Dai, Hiroyuki Morita, Masashi Toyoda, Shuji Takada, Masashi Ikeuchi, Katsuhisa Matsuura, Akihiro Umezawa, Hiroyuki Aburatani, Kenji Onoue, Seitaro Nomura, Norihiko Takeda, Issei Komuro

**Affiliations:** a Department of Cardiovascular Medicine, Graduate School of Medicine, The University of Tokyo, Bunkyo-ku, Tokyo, Japan; b Department of Frontier Cardiovascular Science, Graduate School of Medicine, The University of Tokyo, Bunkyo-ku, Tokyo, Japan; c Center for Epidemiology and Preventive Medicine, The University of Tokyo Hospital, Bunkyo-ku, Tokyo, Japan; d Center for Regenerative Medicine, National Center for Child Health and Development Research Institute, Setagaya-ku, Tokyo, Japan; e Department of Systems Developmental Biology, National Center for Child Health and Development Research Institute, Setagaya-ku, Tokyo, Japan; f Department of Biodesign, Division of Biofunctional Restoration Institute of Biomaterials and Bioengineering, Science Tokyo, Chiyoda-ku, Tokyo, Japan; g Institute of Advanced Biomedical Engineering and Science, Tokyo Women's Medical University, Shinjuku-ku, Tokyo, Japan; h Genome Science Division, Research Center for Advanced Science and Technology, The University of Tokyo, Meguro-ku, Tokyo, Japan; i Department of Cardiovascular Medicine, Nara Medical University, Kashihara-shi, Nara, Japan; j International University of Health and Welfare, Minato-ku, Tokyo, Japan

**Keywords:** dilated cardiomyopathy, DNA damage, iPS cell-derived cardiomyocyte, lamin A/C, vitamin D

## Abstract

**Background:**

In heart failure (HF), DNA damage caused by various external stressors contributes to cardiac dysfunction through the activation of DNA damage response pathways. To date, no clinical strategies have been established to restore cardiac function by reducing accumulated DNA damage. We previously found that vitamin D improved contractility in lamin A/C (*LMNA*) p.Q353R–mutant induced pluripotent stem (iPS) cell-derived cardiomyocytes (iPSCMs), but whether this effect extends to other *LMNA* variants and in vivo models remained uncertain.

**Objectives:**

The objective of the study was to evaluate the association of vitamin D pathway activation with cardiomyocyte phosphorylated histone H2AX (γH2AX) foci and contractile phenotypes in patient-derived iPSCMs and mouse models of HF.

**Methods:**

iPS cell lines were generated from dilated cardiomyopathy patients carrying the *LMNA* p.R225X mutation, and the effects of vitamin D treatment on γH2AX foci and cardiomyocyte contractility were evaluated. In addition, the effects of the vitamin D analog paricalcitol were evaluated in *Lmna* p.R225X mice and in a pressure overload mouse model of HF.

**Results:**

Consistent with previous findings, vitamin D treatment reduced γH2AX foci in cardiomyocytes derived from *LMNA* p.R225X mutant iPS cells through upregulating the expression of DNA repair factors, and improved contractility in these iPSCMs. Furthermore, paricalcitol reduced γH2AX foci and attenuated cardiac dysfunction in both *Lmna* p.R225X mice and pressure overload HF model mice.

**Conclusions:**

Vitamin D pathway activation improved contractile phenotypes across complementary preclinical models and was accompanied by reduced γH2AX foci or related transcriptional changes. These findings support further mechanistic and preclinical investigation.

Although the development of new classes of heart failure (HF) medications has improved outcomes for patients with HF,^1^ the prognosis remains poor, particularly for patients with dilated cardiomyopathy (DCM) associated with lamin A/C (*LMNA*) mutations.^2^ We have recently reported that the accumulation of DNA damage in cardiomyocytes, induced by stimuli such as hypoxia and mechanical stress, is a key mechanism underlying cardiac dysfunction.^3^^,^^4^ Furthermore, we have also reported that the extent of DNA damage accumulation in myocardial biopsy specimens at the time of diagnosis of HF is useful in predicting prognosis^5^ regardless of the underlying disease.^6^ This suggests that in certain cases of HF with significant DNA damage, current guideline-based treatments may be ineffective, leading to disease progression. In addition, the development of new treatments aimed at reducing the accumulation of DNA damage in the heart is considered promising.

Following this concept, in a previous study, we conducted a compound screening using *LMNA*-mutant induced pluripotent stem (iPS) cell-derived cardiomyocytes (iPSCMs), employing DNA damage reduction as a readout, and found that vitamin D2 (VD2) had a beneficial effect.^7^ The mutant iPSCMs treated with VD2 showed increased expression of DNA repair factors, and the contractility of the mutant iPSCMs microtissue was improved. However, this study did not evaluate the effects on the functional phenotype of the heart in an animal model of HF, and it remains uncertain whether the observed improvement in cellular phenotypes contributed to the amelioration of HF. In addition, the study targeted a specific missense mutation of *LMNA* (Q353 R), and it did not confirm whether VD2 is effective against a broad range of primary diseases that cause DNA damage accumulation and HF. Therefore, we sought to determine whether the DNA damage reduction effect of VD2 observed in the previous study could be validated in other cell models and whether it would be effective in animal models.

## Methods

### Patient recruitment, approval from the ethics committee

All the experiments using cells and tissues obtained from patients were approved by the Institutional Review Board of the University of Tokyo Hospital (No. 11044 and No. 11801). Informed consent was obtained from all patients included in this study. All the animal experiments were approved by the University of Tokyo Ethics Committee for Animal Experiments and strictly adhered to the guidelines for animal experiments of the University of Tokyo (approved number P17-058). The protocols for generation of transgenic mice were approved by the Animal Care and Use Committee at the National Research Institute for Child Health and Development, and all experiments were conducted following these approved animal protocols.

### Patient-derived iPSCMs and isogenic control line

Human induced pluripotent stem cells (iPSCs) were generated from peripheral blood mononuclear cells^8^ obtained from a patient with DCM carrying the *LMNA* p.R225X mutation. An isogenic corrected control line was generated using CRISPR/Cas9-mediated genome editing. iPSCs were differentiated into cardiomyocytes using a small molecule-based cardiac differentiation protocol^9^ and metabolically purified before analysis. Detailed procedures for iPSC generation, genome editing, cardiac differentiation, and validation are provided in the Supplemental Methods.

### Assessment of cardiomyocyte phosphorylated histone H2AX foci and contractile force

Cardiomyocyte phosphorylated histone H2AX (γH2AX) foci were assessed by immunostaining for γH2AX and cardiac troponin T, followed by image-based quantification. Contractile force was assessed using engineered cardiac microtissues generated from iPSCMs. Tissue contraction was recorded by live-cell imaginger SI8000, and force was estimated from pillar deflection^10^ as described in the Supplemental Methods.

### Mouse models and paricalcitol treatment

*LMNA* p.R225X knock-in mice were generated using CRISPR/Cas9-mediated genome editing. Pressure overload was induced in male C57BL/6J mice by transverse aortic constriction. Paricalcitol was administered using a weight-based regimen at 5 μg/kg body weight by intraperitoneal injection every other day. Vehicle-treated mice served as controls. Detailed procedures for mouse generation, transverse aortic constriction (TAC) surgery,^11^ drug administration, and biochemical assays are provided in the Supplemental Methods.

### Echocardiography and tissue analysis

Transthoracic echocardiography was performed under isoflurane anesthesia by a blinded operator.^12^ Fractional shortening and additional left ventricular parameters were quantified from 2-dimensional and M-mode images. For tissue analysis, hearts were fixed, sectioned, and stained for γH2AX, wheat germ agglutinin, and 4',6-diamidino-2-phenylindole. Cardiomyocyte γH2AX foci were quantified using animal-level experimental units, with nuclei treated as nested measurements within each animal.

### RNA analysis and statistics

RNA sequencing was performed using isolated cardiomyocytes from mouse hearts, and pathway enrichment analysis was performed for genes upregulated by paricalcitol treatment. Selected DNA repair-related transcripts were validated by quantitative polymerase chain reaction (qPCR). Data are presented as mean ± SD or SEM unless otherwise indicated. Statistical analyses were performed using appropriate tests according to the experimental design, including repeated-measures or mixed-effects models for longitudinal echocardiography and animal-level or nested analyses for histological quantification.

## Results

### *LMNA* p.R225X iPSCMs exhibit increased γH2AX foci and impaired contractility

To validate the efficacy of VD2 in heart disease with a mutation other than the *LMNA* p.Q353 R mutation, we generated iPS cells from a patient with familial DCM carrying *LMNA* p.R225X mutation and differentiated them into cardiomyocytes for analysis.^13^ iPS cell lines were established from the peripheral blood mononuclear cells of a 65-year-old female patient (Figure 1A), and an isogenic control line was generated by repairing the corresponding mutation to the wild type using CRISPR/Cas9 gene editing for comparison (Figure 1B). Using differentiated iPSC-CMs, we assessed cardiomyocyte nuclear γH2AX foci by immunostaining. *LMNA* p.R225X-mutant cardiomyocytes showed significantly increased γH2AX foci compared with isogenic corrected cardiomyocytes, indicating increased accumulation of a nuclear DNA damage response (DDR) marker (Figures 1C and 1D). We next generated cardiac microtissues from iPSCMs and examined the tension of spontaneous contraction of the tissue (Figure 1E). The estimated force of mutant iPSCMs with *LMNA* p.R225X was significantly lower than that of the corrected iPSCMs (Figure 1F). In summary, the mutant iPSCMs exhibited increased γH2AX foci and reduced contraction force compared to the corrected iPSCMs, indicating successful disease modeling.

**Figure 1:**
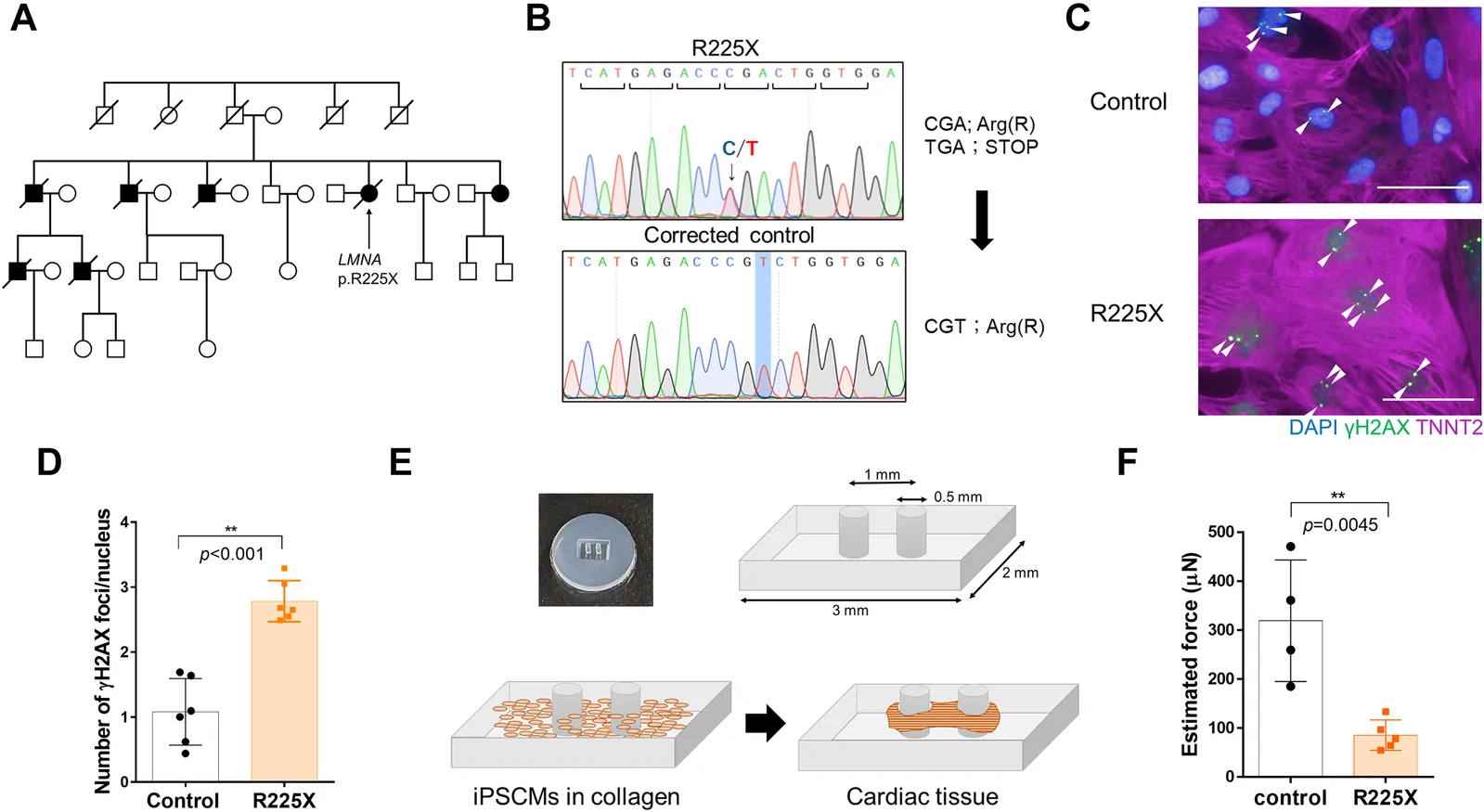
*LMNA* p.R225X iPSCMs Show Increased γH2AX foci (A) Pedigree with the *LMNA* p.R225X mutation. Arrow indicates patient from whom iPS cells were established. (B) Sanger sequencing of the *LMNA* p.R225X mutant and genome-edited isogenic control lines. (C) γH2AX and TNNT2 immunostaining. Arrow heads indicate γH2AX foci. Scale bar: 200 μm. (D) γH2AX-positive foci per iPSCM nucleus. Dots represent 6 well-level replicates from 2 independent differentiation batches. (E) Microtissue device (top) and formation scheme (bottom). (F) Contractile tension of corrected (n = 4) and mutant (n = 5) microtissues. Each point represents 1 microtissue from 1 differentiation batch. Data are mean ± SEM. Two-sided unpaired Student’s t-tests were used in D and F. ∗∗*P* < 0.01. CGA = cytosine-guanine-adenine codon (arginine); CGT = cytosine-guanine-thymine codon (arginine); DAPI = 4',6-diamidino-2-phenylindole; iPSCM = induced pluripontent stem cell-derived cardiomyocyte; LMNA = lamin A/C gene; TGA = thymine-guanine-adenine stop codon; TNNT2 = cardiac troponin T; γH2AX = phosphorylated histone H2AX.

### VD2 reduces γH2AX foci and improves contractile force in *LMNA* p.R225X-mutant iPSCM

We investigated whether VD2 exerted similar effects in mutant iPSCMs harboring the *LMNA* p.R225X mutation, as had been observed in *LMNA* p.Q353R-mutant iPSCMs in our previous study. VD2 treatment reduced γH2AX-positive nuclear foci in *LMNA* p.R225X-mutant iPSCMs in a concentration-dependent manner (Figure 2A). Immunostaining for vitamin D receptor (VDR) revealed increased nuclear localization of VDR following VD2 treatment (Figure 2B). To determine whether the vitamin D-responsive transcriptional program identified in our previous *LMNA* p.Q353 R model was also relevant to the *LMNA* p.R225X model, we reanalyzed RNA-seq data from our previous study of p.Q353R-mutant cardiomyocytes before and after VD2 treatment (GSE182219).^7^ We observed that many DNA repair genes were consistently upregulated in 2 independent iPSCM clones following VD2 administration (Figure 2C). When we examined the expression changes of these gene groups after VD2 treatment using qPCR, we found that these gene sets were also increased at higher levels in the p.R225X-mutant iPSCMs (Figure 2D). Thus, the RNA-seq reanalysis supports a shared vitamin D-responsive repair-related transcriptional program, whereas direct transcriptional validation in the p.R225X model was provided by qPCR. In addition, cardiac microtissues generated from p.R225X iPSCMs exhibited improved contractility following VD2 administration (Figure 2E). These findings indicate that VD2 was associated with reduced γH2AX foci and improvement of cardiomyocyte contractility in *LMNA* p.R225X iPSCMs.

**Figure 2:**
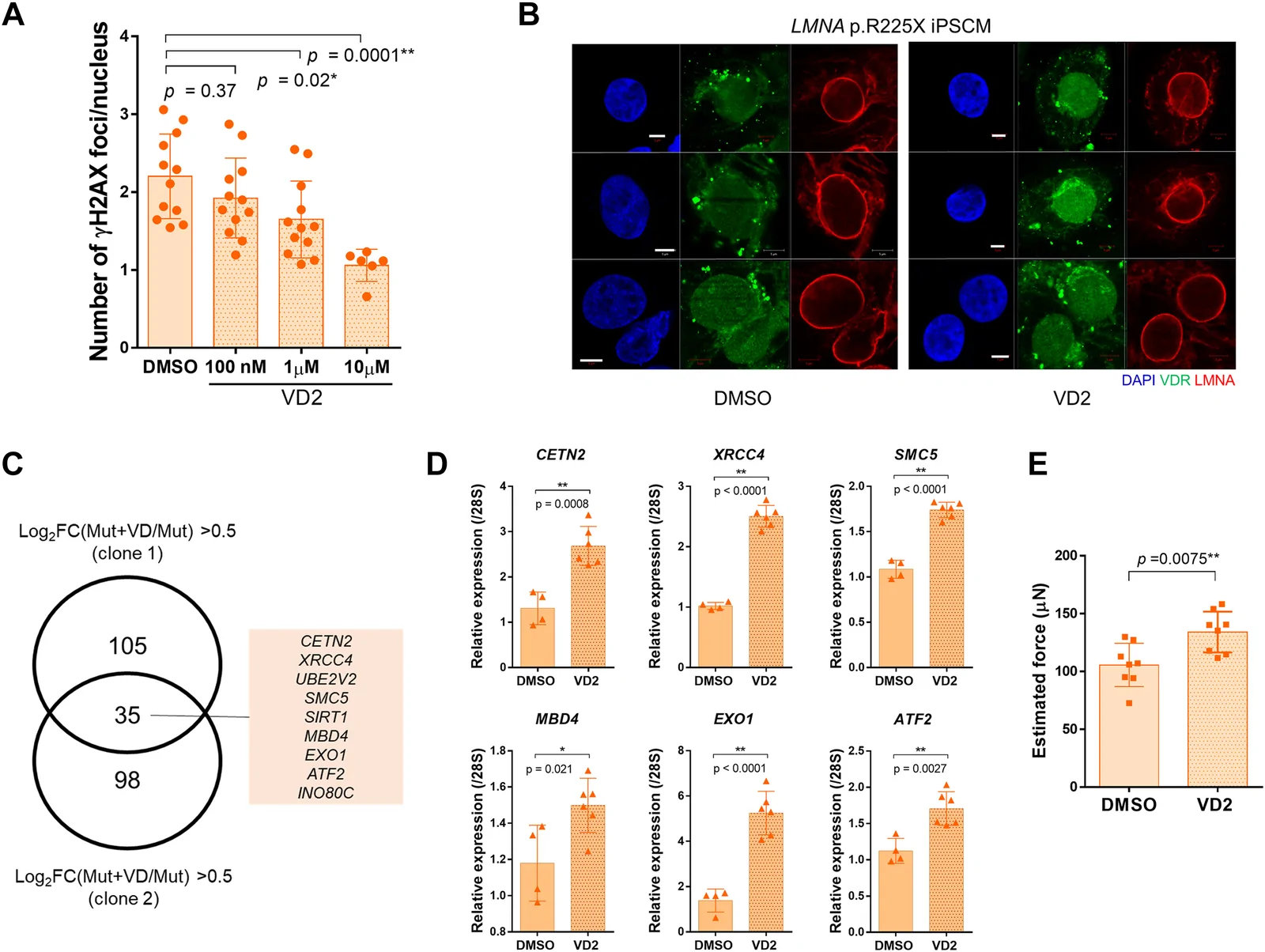
VD2 Improves *LMNA* p.R225X iPSCM Phenotypes (A) γH2AX-positive foci in *LMNA* R225X iPSCMs treated with vitamin D2 (VD2). (B) Vitamin D receptor (VDR) immunostaining after VD2 or vehicle treatment. Scale bar, 5 μm. (C) DNA repair factors upregulated by VD2 in Q353 R iPSCMs, based on reanalysis of GSE182219. (D) qPCR of selected transcripts in *LMNA* p.R225X iPSCMs treated with VD2 (n = 6) or vehicle (n = 4). Dots represent samples from 2 independent differentiation batches. (E) Contractile tension of vehicle- and VD2-treated microtissues (n = 8 per group). Each point represents 1 microtissue from 1 differentiation batch. Data are mean ± SEM; A, 1-way ANOVA with Tukey multiple-comparisons; D and E, 2-sided unpaired Student’s t-tests. ∗*P* < 0.05; ∗∗*P* < 0.01. DMSO = dimethyl sulfoxide; log2FC = log2 fold change; Mut = mutant; qPCR = quantitative polymerase chain reaction; VD = vitamin D; other abbreviations as in Figure 1.

### Paricalcitol reduces cardiomyocyte γH2AX foci and preserves systolic function in *LMNA* p.R225X mice

We next evaluated the efficacy of VD2 in an animal model of HF carrying a DCM-associated mutation. As mice with the heterozygous p.Q353 R mutation, the target of the previous work, were perinatal lethal, it was difficult to assess cardiac function. Therefore, we established a mouse model with the homologous *LMNA* p.R225X/WT mutation, corresponding to the iPSCMs described in the previous section (Figure 3A). In the hearts of these mice, cardiomyocyte nuclear γH2AX positivity was greater than that in wild-type mice at 3 months of age (Figures 3B and 3C). Paricalcitol-treated animals exhibited numerically lower cardiomyocyte γH2AX foci than vehicle-treated mice; however, this difference did not reach statistical significance in the nested animal-level analysis (Figures 3B and 3C). Echocardiography revealed a gradual decline in cardiac function beginning at 8 weeks in the mutant mice, whereas paricalcitol-treated mice showed higher fractional shortening over the observation period, with a trend toward attenuation of the temporal decline in systolic function (Figure 3D, Supplemental Figure 1A). Cardiac fibrosis and cardiomyocyte cross-sectional area were not markedly changed by paricalcitol (Supplemental Figures 2A and 2B). To assess potential biochemical adverse effects of paricalcitol treatment, we measured plasma calcium and inorganic phosphate levels in vehicle- and paricalcitol-treated mice. They were not significantly different between the 2 groups (Supplemental Table 1). These data collectively suggest that activation of vitamin D pathway is associated with reduced γH2AX foci in cardiomyocytes and improve cardiac function during short-term follow-up in this *LMNA* cardiomyopathy mouse model.

**Figure 3:**
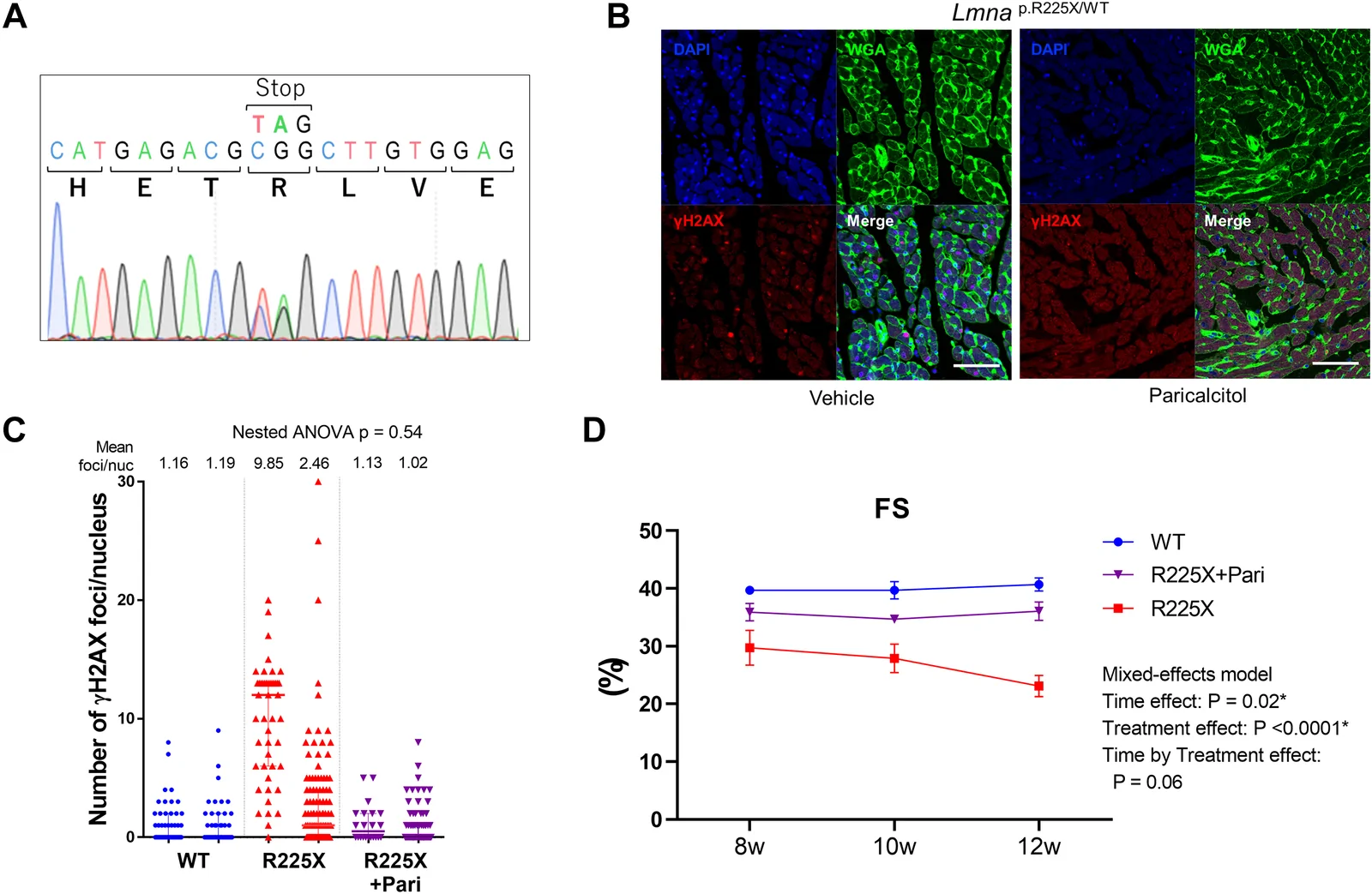
Paricalcitol Preserves Cardiac Function of *Lmna* p.R225X Mice (A) Sanger sequencing of the *Lmna* R225X/WT allele. (B) Myocardial γH2AX staining in 12-week-old *Lmna* R225X mice with and without paricalcitol. Scale bar, 100 μm. (C) Cardiomyocyte nuclear γH2AX foci; nuclei are nested within mice (n = 2 mice per group). (D) Serial fractional shortening in wild-type (n = 3) and *Lmna* R225X (n = 7), and R225X + paricalcitol (n = 4) mice. Two measurements in R225X group at 10 and 12 weeks, and 1 measurement in the R225X + paricalcitol group at 12 weeks, were unavailable because of planned tissue collection. Data are mean ± SEM; C, nested 1-way ANOVA. (D) REML mixed-effects model with group, categorical time, group × time interaction, mouse random intercept, Geisser-Greenhouse correction. ∗*P* < 0.05; ∗∗*P* < 0.01. ANOVA = analysis of variance; Pari = paricalcitol; WGA= wheat germ agglutinin; WT = wild type; other abbreviations as in Figure 1.

### Paricalcitol attenuates γH2AX foci accumulation and systolic dysfunction after pressure overload

To assess whether VD2 is effective in preventing γH2AX foci accumulation and cardiac dysfunction due to etiologies other than *LMNA* mutations, a VD2 analog (paricalcitol) was administered to mice with pressure-overload HF induced by TAC. Previous reports have demonstrated the accumulation of DNA damage in cardiomyocytes of failing hearts following TAC.^3^ Mice were administered paricalcitol simultaneously with TAC-induced pressure overload, followed by histological and echocardiographic evaluations of cardiac function (Figure 4A). After 4 weeks of TAC, the paricalcitol-treated group exhibited tendency toward reduction of γH2AX foci accumulation compared to the vehicle group (Figures 4B and 4C). After 6 weeks of TAC, the paricalcitol-treated group demonstrated a significantly attenuated decline in cardiac function and a reduction in cardiac weight (Figures 4D and 4E, Supplemental Figure 1B). Treatment with paricalcitol tended to reduce cardiac fibrosis, but there was no change in cardiomyocyte cross-sectional area (Supplemental Figures 2C and 2D). To clarify the mechanism by which paricalcitol exerts its efficacy, we isolated cardiomyocytes from the mouse hearts and performed RNA sequencing. Gene ontology analysis of genes upregulated in the paricalcitol-treated group revealed enrichment of multiple biological pathways, including mitochondrial function, cellular homeostasis, and DNA repair-related pathways (Figure 4F). We examined the expression of the DNA repair factors that were upregulated after VD2 administration in the iPSCMs by qPCR and found that they were significantly upregulated by paricalcitol administration (Figure 4G). These results suggest that paricalcitol treatment was associated with reduced cardiomyocyte γH2AX foci, induction of DNA repair-related transcripts, and attenuation of systolic dysfunction in pressure overload-induced cardiac remodeling.

**Figure 4:**
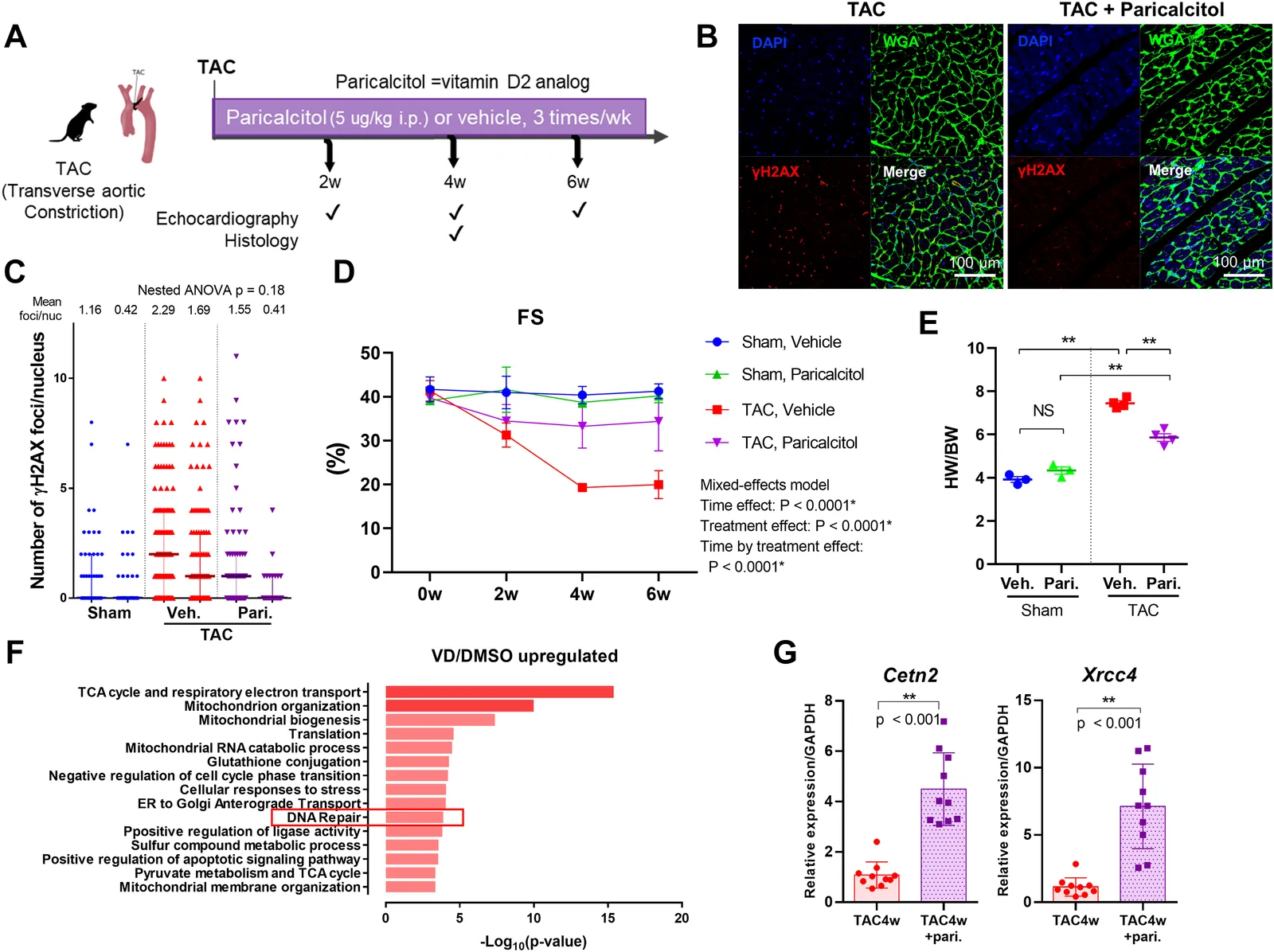
Paricalcitol Attenuates TAC-Induced γH2AX Foci and Cardiac Dysfunction (A) Experimental design. Paricalcitol or vehicle was administered every other day from day 2 after transverse aortic constriction (TAC) through week 6. (B) Myocardial γH2AX staining. Scale bar, 100 μm. (C) Cardiomyocyte nuclear γH2AX foci; nuclei are nested within mice (n = 2 per group). (D) Serial fractional shortening in sham (n = 6) and TAC (n = 6), sham + paricalcitol (n = 3), and TAC + paricalcitol (n = 8) mice. Four measurements in the TAC group at 6 weeks were unavailable because of planned tissue collection. (E) Heart weight-to-body weight ratio at week 6. sham, n = 3; TAC, n = 4. (F) Gene ontology term enrichment analysis of the top 100 genes up-regulated by paricalcitol after TAC. (G) qPCR of DNA repair factors in TAC mouse hearts (n = 10). Data are mean ± SEM. (C) Nested 1-way ANOVA; (D) restricted maximum likelihood mixed-effects model with categorical time, group, interaction, mouse random intercept, Geisser-Greenhouse correction; (E) 1-way ANOVA with Tukey comparisons; (G) 2-sided unpaired Student’s t-test. ∗*P* < 0.05; ∗∗*P* < 0.01. BW = body weight; ER = endoplasmic reticulum; GAPDH = glyceraldehyde-3-phosphate dehydrogenase; HW = heart weight; NS = not significant; qPCR = quantitative polymerase chain reaction; TCA = tricarboxylic acid; other abbreviations as in Figures 1 and 3.

## Discussion

In conjunction with previous findings, the current study shows that vitamin D pathway activation was associated with reduced cardiac γH2AX foci and improved contractile phenotypes across 3 preclinical models: missense and nonsense mutations in *LMNA*, and pressure overload induced by TAC. To date, no therapeutic agents have been developed to mitigate DNA damage in cardiomyocytes.

Vitamin D is mainly synthesized in the skin from 7-dehydrocholesterol by ultraviolet irradiation,^14^ so it makes sense that vitamin D would induce a group of molecules that respond to DNA damage,^15^ considering that ultraviolet rays can cause cancer in the skin by damaging DNA and inducing genomic mutations. In fact, studies in cancer cells have reported that vitamin D contributes to genomic integrity by maintaining the stability of repair molecules such as p53-binding protein 1 (53BP1) and breast cancer 1 protein.^16^

The DDR generally exerts beneficial effects on organs by eliminating dysfunctional cells, primarily through inducing growth arrest or apoptosis in cells with extensively damaged genomes.^17^^,^^18^ Therefore, suppression of downstream effectors of the DDR, such as p53 and p21,^19^^,^^20^ may have systemic deleterious effects, including an increased risk of carcinogenesis. Vitamin D is expected to reduce both upstream DNA damage and downstream DDRs by inducing DNA repair factors.^21^^,^^22^ This mechanism is considered more physiologically favorable and may also have potential for future applications in the treatment of aging.^23^

There have been several clinical trials of vitamin D in patients with HF. The VINDICATE trial was a randomized control trial in which vitamin D was given to 200 patients with HF and vitamin D deficiency.^17^ Although no improvement in physical function was observed after 1 year of treatment, significant improvements in cardiac function and reversal of left ventricular remodeling were reported. In the EVITA trial,^24^ more than 400 patients were administered vitamin D3 for 3 years. Although no improvement in HF-related mortality was observed, a subsequent subanalysis demonstrated improved cardiac function in patients over 50 years of age.^25^ In addition, a study using Japanese medical records showed that in an analysis of over 10,000 patients hospitalized for acute HF, the prognosis was better in the group of patients who were taking vitamin D at the time of hospitalization.^26^ These studies suggest that vitamin D is effective for HF; however, vitamin D may increase calcium levels and promote vascular calcification, leading to a negative hemodynamic impact in terms of atherosclerosis and reduced vascular stiffness.^27^ In the present study, plasma calcium and inorganic phosphate levels were not significantly increased by paricalcitol treatment; however, plasma paricalcitol concentrations, circulating vitamin D metabolites, renal function, and histological assessment of vascular calcification were not evaluated. Future studies should include pharmacokinetic and pharmacodynamic analyses and systematic safety assessments to define the therapeutic window of vitamin D pathway activation in cardiac disease models. It is necessary to consider methods to select appropriate responders. Targeting patients with high levels of cardiomyocyte DNA damage, as determined by biopsy, may improve the efficacy of vitamin D–based therapies.

Caution is required when interpreting γH2AX foci in LMNA cardiomyopathy. LMNA are major structural components of the nuclear lamina and contribute to maintaining nuclear membrane integrity, chromatin organization, nuclear mechanical signaling, and the damage response, including DNA repair. Therefore, LMNA mutations may influence γH2AX foci through mechanisms that go beyond simply an increased DNA damage burden. For example, they may alter the accessibility of various molecules to chromatin, the spatial arrangement of damaged chromatin, the mobilization or retention of DDR factors, and the dynamics of γH2AX focus formation and disappearance, potentially further amplifying nuclear stress and DDR signaling in cardiomyocytes subjected to mechanical stress. Similarly, the reduction in γH2AX foci following administration of VD2 or paricalcitol may reflect a reduction in DNA damage burden, changes in DDR signaling, improved nuclear homeostasis, or promotion of γH2AX focus resolution. This study directly demonstrates that activation of the vitamin D pathway is associated with a reduction in γH2AX foci, induction of DNA repair-related transcripts, and improved contractile function in cardiomyocytes. However, this study does not directly prove that activation of the vitamin D pathway repairs DNA damage or that the reduction in γH2AX foci causally mediates the improvement in contractile function. To elucidate these mechanisms, further studies are needed using orthogonal DNA damage assays, live-cell analyses of nuclear envelope integrity, and direct assessments of DDR factor recruitment and focus resolution.

This study extends our previous findings to additional in vitro and in vivo preclinical models. The results support further investigation of vitamin D pathway activation in HF subsets characterized by increased cardiomyocyte DDR. Additional validation studies in larger preclinical cohorts are required before clinical application can be considered (Central Illustration).

**Central Illustration:**
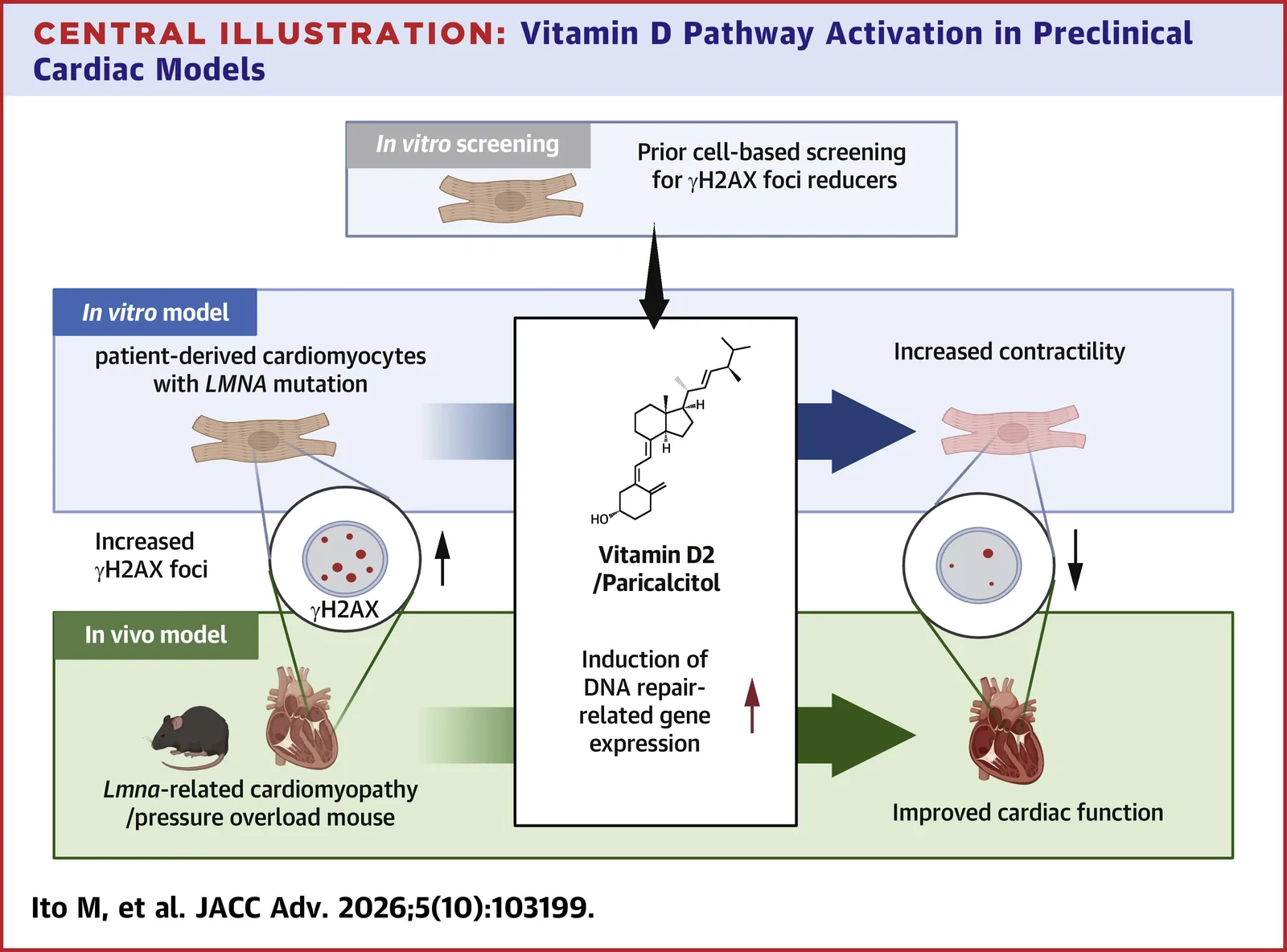
Vitamin D Pathway Activation in Preclinical Cardiac Models Patient-derived cardiomyocytes carrying an *LMNA* mutation and mouse models of *Lmna*-related cardiomyopathy or pressure overload were used to evaluate vitamin D pathway activation. Vitamin D2 or paricalcitol was associated with fewer cardiomyocyte γH2AX foci, a marker of DNA damage response, increased expression of DNA repair-related genes, improved contraction of engineered heart muscle tissues and preservation of systolic function in mouse models. These findings lay the groundwork for future research on the potential role of vitamin D in the prevention and treatment for heart failure. LMNA, lamin A/C gene; γH2AX, phosphorylated histone H2AX.

### Study limitations

The present study should be interpreted as demonstrating an association, rather than a definitive causal relationship, between vitamin D pathway activation and reduced cardiomyocyte γH2AX foci, induction of DNA repair factors, and improved contractile phenotypes. Although reduced γH2AX foci were consistently observed in VD2- or paricalcitol-treated models, these data do not prove that attenuation of cardiomyocyte DDR is the direct mechanism responsible for improved contractile function. Although γH2AX is widely used as a marker of DDR, it can also reflect altered chromatin signaling and DDR activation as well as DNA lesion burden. Therefore, reduced γH2AX foci after VD2 or paricalcitol treatment should not be interpreted as definitive evidence of repaired DNA or reduced DNA lesions. Future studies incorporating 53BP1 colocalization, comet assays, direct measurements of DNA repair activity, and broader DDR pathway analyses will be necessary to determine whether vitamin D pathway activation directly reduces DNA lesion burden or primarily modulates DDR signaling.

VDR activation can affect multiple biological pathways relevant to cardiac function, including mitochondrial homeostasis, calcium handling, oxidative stress, and inflammatory signaling. Indeed, transcriptomic analysis in pressure-overloaded hearts showed enrichment of both DNA repair- and mitochondrial function-related pathways after paricalcitol treatment. Therefore, the observed functional improvement may reflect the combined effects of vitamin D pathway activation on several cardiomyocyte and noncardiomyocyte processes. Future studies using direct genetic or pharmacological manipulation of DNA repair pathways will be necessary.

There are several technical concerns regarding the experiments in this study. The in vitro experiments using *LMNA* p.R225X iPSCMs were conducted using a single patient-derived line and its isogenic correction control line, and the number of independent differentiation batches was limited. Therefore, these results should be interpreted as insights into the mechanisms underlying this specific genetic background and reproducibility must be verified using samples from multiple cell lines and differentiation batches.

In vivo echocardiography and histological analysis were limited by the small number of animals and the relatively short follow-up period after drug administration, as well as observing parameters. Although consistent effects of vitamin D pathway activation were observed within the planned experiments, this study cannot conclusively determine whether the therapeutic effects are robust in both mouse models, or whether activation of the vitamin D pathway can sustainably prevent the progression of HF and long-term adverse cardiac remodeling. Additional studies involving independent larger cohorts and continuous functional assessments over long-term follow-up are required. In addition, the assessment of in vivo cardiac function was primarily based on echocardiographic parameters, which are influenced by loading conditions. Heart rate was not significantly different between vehicle- and paricalcitol-treated mice (Supplemental Figure 1). However, systemic blood pressure and invasive hemodynamics such as trans-stenotic gradients were not measured. Therefore, we cannot exclude the possibility that differences in afterload or other loading conditions contributed to the observed echocardiographic findings. Thus, future studies incorporating Doppler assessment of aortic constriction severity, blood pressure monitoring and pressure-volume loop analysis will be required to determine whether vitamin D pathway activation improves load-independent indices of myocardial contractile function under comparable pressure overload.

In the histological analysis, fibrosis and cardiomyocyte size was not significantly changed by paricalcitol treatment in either model, suggesting that the preservation of systolic function by paricalcitol cannot be explained by reduced fibrosis or cardiomyocyte hypertrophy alone. Histological evaluations including apoptosis levels, capillary density, or more detailed ultrastructural changes will be necessary to understand precise molecular mechanisms of vitamin D pathway activation.

Perspectives **COMPETENCY IN MEDICAL KNOWLEDGE:** Cardiomyocyte DDR may contribute to contractile dysfunction in selected forms of HF. In preclinical models, activation of the vitamin D pathway was associated with improved contractile phenotypes, reduced cardiomyocyte γH2AX foci, and induction of DNA repair-related transcripts.

**TRANSLATIONAL OUTLOOK:** Further studies are needed to determine whether vitamin D pathway activation causally improves cardiac function through modulation of cardiomyocyte DDR, to identify responsive HF subsets, and to establish optimal dosing and safety profiles, including calcemic and vascular effects.

## Funding support and author disclosures

This study was partly funded by Grant-in-Aid for Young Scientists (to Drs Ito and Katoh), Grant-in-Aid for Scientific Research (C) (to Drs Ito and Katoh), (A) (to Dr Nomura), and (S) (to Dr Komuro) from Japan Society for the Promotion of Science; the SENSHIN Medical Research Foundation, Japan (to Dr Ito); the Japan Foundation for Applied Enzymology (to Dr Ito); the Japan Heart Foundation (to Drs Ito and Katoh); A Japanese Circulation Society grant for basic research (2019, 2022) (to Dr Ito); the KANAE medical research foundation (to Dr Ito); the Takeda Science Foundation (to Dr Ito); the MSD Life Science Foundation (to Dr Ito); research funds from Tsurugaya Hospital (to Dr Ito); a Grand-in-aid of the Cardiovascular Research Fund (to Dr Ito); the Mochida Memorial Foundation for Medical and Pharmaceutical Research (to Dr Ito); the UTEC-UTokyo FSI Research Grant Program (to Dr Nomura); the JST FOREST Program (Grant Number JPMJFR210U) (to Dr Nomura); 10.13039/100009619Japan Agency for Medical Research and Development grants; JP16bm0609004, JP19bm0804010, JP22gm0810013, JP22ek0109440, JP20ek0210152, JP20ek0109440, JP18km0405209, 10.13039/100009619JP21ek0109543, JP22ama121016, JP22ek0210172, JP22ek0210167, JP22bm1123011, JP23gm4010020, JP22ek0109617, JP23tm0524009, JP23tm0524004, JP23jf0126003, JP24ek0109755, JP24ek0210205, JP24jf0126011, JP25bk0104192 and JP25ek0109795 to Drs Nomura and Komuro, JP23bm1423007h0001 and JPbm1423026h0001 to Dr Ito. All other authors have reported that they have no relationships relevant to the contents of this paper to disclose.
