# Supplementary material for: Vitamin D Pathway Activation Reduces Cardiomyocyte DNA Damage and Improves Cardiac Contractility in Preclinical Models
Source: JACC Adv. 2026 Sep 9;5(10):103199. doi: 10.1016/j.jacadv.2026.103199 (PMC13699713; doi:10.1016/j.jacadv.2026.103199)
Supplement: Supplementary file 1 [file mmc1.pdf]

Supplementary Fig.1

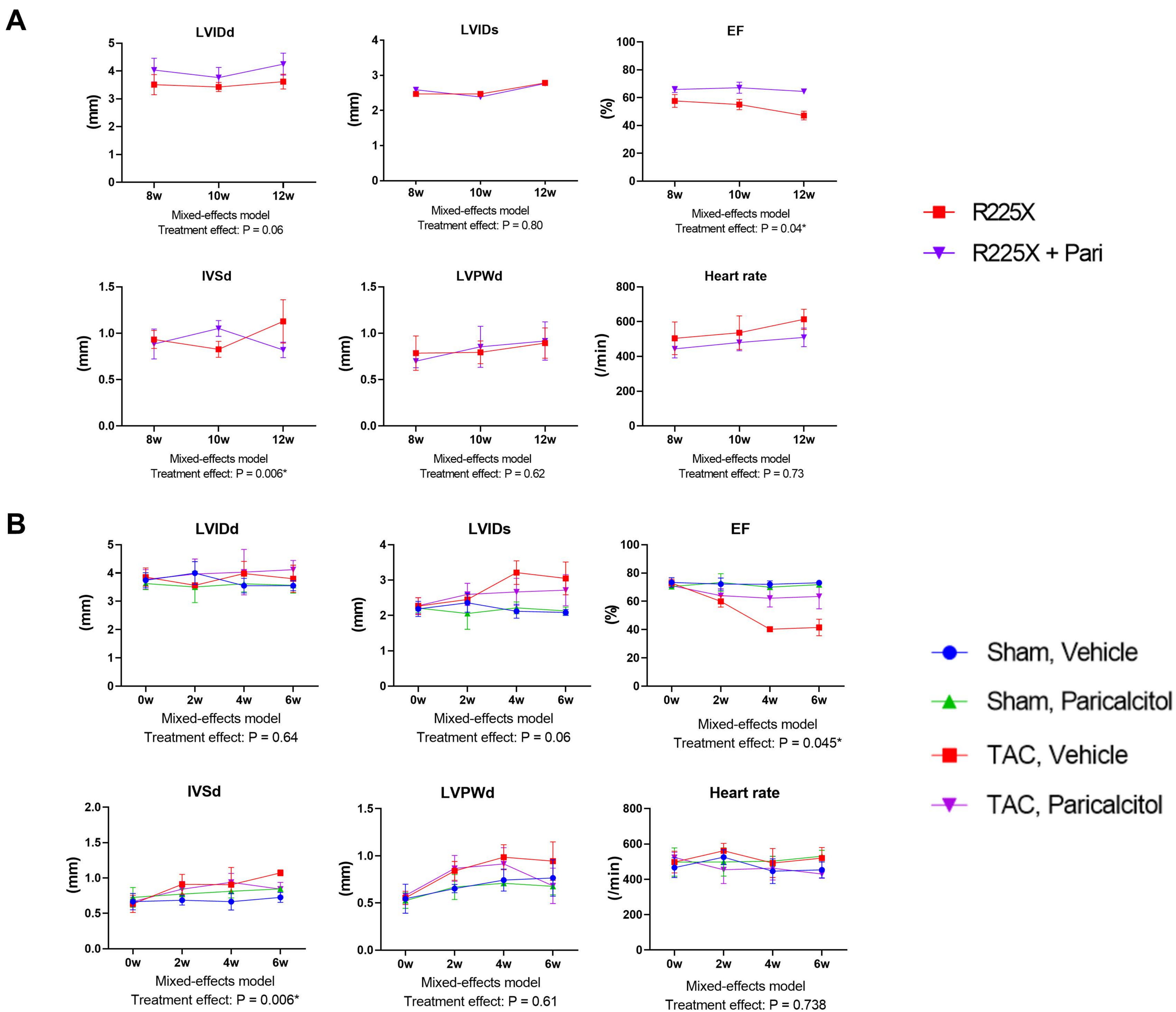

Supplementary Fig.2

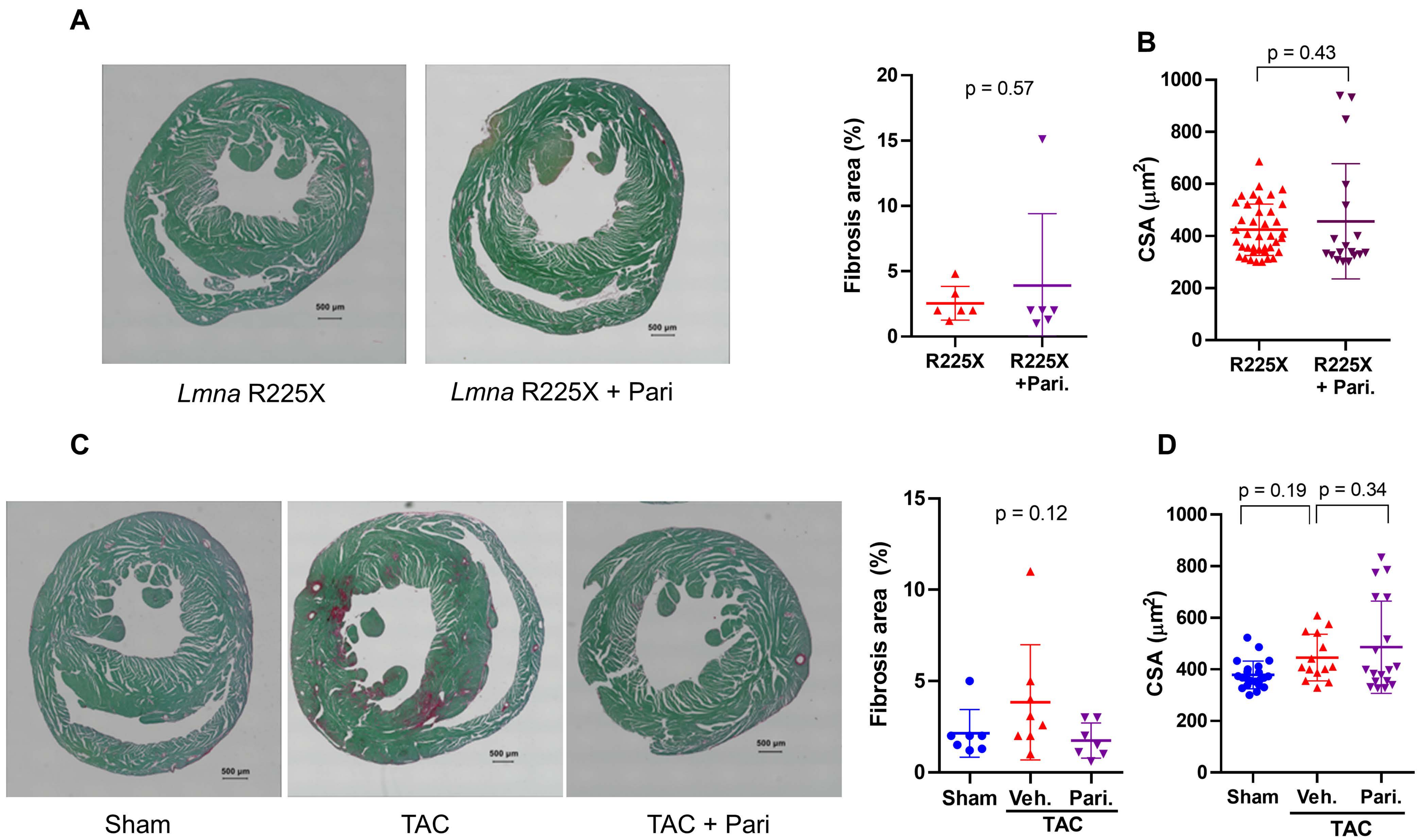

Supplementary Figure 1. Additional echocardiographic parameters in *Lmna* p.R225X and TAC mice.

Diastolic and systolic left ventricular internal dimensions (LVIDd/s), Left ventricular ejection fraction (EF), diastolic interventricular septal thickness (IVSd) , diastolic posterior wall thickness (LVPWd) , and heart rate were measured by transthoracic echocardiography. (A) *Lmna* R225X mice, related to Fig. 3D. n = 7 (R225X), 4 (R225X + Pari). (B) TAC mice, related to Fig. 4D. n = 6 (sham), 6 (TAC), 3 (sham + Pari), 8 (TAC + Pari).

Pari, Paricalcitol; TAC, transverse aortic constriction. Data are shown as mean ± SD with individual animal values.

Supplementary Figure 2. Additional histological analysis in *Lmna* p.R225X and TAC mice.

Picro-sirius staining images of heart, assessment of myocardial fibrosis, and cardiomyocyte cross-sectional area in *Lmna* p.R225X (A, B) and TAC mice (C, D), with or without paricalcitol treatment. (A) n (slides) = 6 from two different mice per group. (B) n (view filed) = 22, 14, 18 in 6 slides, from two different mice per group. (C) n (slides) = 7 from two different mice per group. (D) n (view filed) = 40, 18 in 7 slides, from two different mice per group.

Supplementary Methods

1. Patient recruitment, approval from the ethics committee

All the experiments using cells and tissues obtained from patients were approved by the Institutional Review Board (IRB) of the University of Tokyo Hospital (No. 11044 and No. 11801). Informed consent was obtained from all patients included in this study. All the animal experiments were approved by the University of Tokyo Ethics Committee for Animal Experiments and strictly adhered to the guidelines for animal experiments of the University of Tokyo (approved number P17-058). The protocols for generation of transgenic mice were approved by the Animal Care and Use Committee at the National Research Institute for Child Health and Development, and all experiments were conducted following these approved animal protocols.

2. Creation of iPS cells, induction of cardiomyocyte differentiation

iPS cells derived from the patients were established using Sendai virus (SeV) vectors encoding human OCT3/4, SOX2, KLF4, and c-MYC (CytoTune®-iPS 2.0, ID Pharma). Briefly, 2×10^5 to 8×10^5 peripheral blood mononuclear cells (PBMCs) separated from whole blood using Ficoll-Paque PLUS (Cytiva) were infected with SeV vectors (MOI = 2) for 2 h. The infected PBMCs were seeded on mitomycin C-treated mouse embryonic feeder cells (EmbryoMax, PMEF CF1, Millipore) and were cultured in KBM501 (Kojin Bio) containing 10% fetal bovine serum (FBS) for two days. The cells were cultured in iPSellon medium (Cardio) supplemented with 10 ng/mL basic FGF (Thermo Fisher) for 10-20 days. Then, iPS cell colonies were isolated and maintained on feeder cells. Before cardiac differentiation, they were transferred to a feeder-free culture system, cultured on Vitronectin-N (Thermo Fisher)-coated dishes, fed with Essential 8 Flex medium (Thermo Fisher) at 37° C and 5% (v/v) CO_2, and passaged with 0.5 mM EDTA solution (Nacalai Tesque).

3. Cardiac differentiation of iPS cells

Subconfluent iPS cells were differentiated into cardiomyocytes as described previously. On day 0, the medium was replaced with the AscleStemTM cardiomyocyte differentiation medium kit (CDM, Nacalai Tesque) containing 6 μM CHIR99021 (TOCRIS). On day 2, the medium was replaced with CDM supplemented with 5 μM IWP-2 (TOCRIS) and 5 μM SB431542 (Wako). After day 6, the medium was replaced with CDM every other day. On day 10, cells were dissociated into single cells using Liberase solution (Roche), followed by TryPLE Express (Thermo Fisher). On day 10, the cells were purified using DMEM without glucose and glutamine (Life Technologies) containing 4 mM lactate (Wako). The non-

glucose lactate medium was exchanged every two days for 5 days. After purification, the cells were fed with DMEM containing 10% FBS (Thermo Fisher). On day 28, the cardiomyocytes were cryopreserved in CELLBANKER 1 plus solution (Nippon Zenyaku) using a controlled rate deep freezer (STREX Inc.). The stocks were thawed and maintained with DMEM with 10% FBS and then used for analysis around day 35. Active form of vitamin D2 was diluted in DMSO and used for in vitro experiments.

4. Gene editing of iPS cells

Both gRNAs and single-stranded oligodeoxynucleotides (ssODNs) for iPS cell gene editing were designed using CRISPRdirect (https://crispr.dbcls.jp/). The PAM sequences were selected to contain the mutated base so that only mutant allele was targeted. Then, gRNAs were constructed using the Guide-it sgRNA In Vitro Transcription Kit (Takara). All sgRNA, Cas9 protein (Takara), and ssODN constructs were delivered into iPS cells by nucleofection using NEPA21 (NEPA gene). After cloning transfected iPS cells, the established clones were genotyped by PCR and bidirectional direct sequencing. Sequences of gRNA and ssODN for R225X mutation knock-in are as follows.

gRNA; GCGCCGTCATGAGACCtGACTGG ('t' is the corresponding mutation site)

ssODN: 5'-AACTCACGCTGCTTCCCATTGTCAATCTCCACCAGACgGGTCTCATGACGGCGCTTGGTCTCACGCAGCTCCT -3'

5. Creation of myocardial tissue and measurement of tension

The culture device for microtissue formation was sculpted using PDMS; the device consists of two cylinders 500 μm in diameter and 1 mm high, spaced 500 μm apart, in a tub 2 × 3 mm square and 500 μm deep, and was manufactured with Ecoflex (Ecoflex 00-20, SMOOTH-ON Inc.). Briefly, the precision aluminum mold for the devices was fabricated using a 3-D milling machine. The surfaces were dip coated with a thin layer of fluoropolymer (CYTOP, AGC) to enhance mold release. Uncured polymer solutions of Ecoflex were mixed, injected into the mold, defoamed in a vacuum chamber, and allowed to cure at room temperature overnight. Then, the devices were removed from the mold and irradiated with excimer ultraviolet light (172 nm, E172-110, Excimer Inc., Japan) for 15 s to hydrophilize the surfaces. The devices were coated with 3% BSA, and then tissue formation was performed with cell solution suspended in collagen gel. A solution of bovine acid-solubilized collagen I (FujiFilm Wako, final concentration 2.4 mg/mL), 10x MEM medium (10%, v/v), and Matrigel (BD, final concentration 10%, v/v) was mixed on ice and neutralized with NaOH. Cellular density was adjusted to contain 5.0×10^4 cells/3 μL of suspension per microtissue, and the solution was

placed in molds pre-coated with 3% BSA solution and maintained at 37° C for 45 min while maintaining saturated water vapor pressure. After gel formation, cells were cultured in DMEM medium containing 10% FBS, and the medium was changed every 2 days. The cell suspension gels contracted to bridge the columns and began to beat synchronously in about 48 h. Tissue contraction kinetics were evaluated after 1 week, and the effect of vitamin D2 was assessed at 2 weeks, with administration beginning at 1 week. After tissue preparation, the beating was filmed for 10 s using the Live Cell Imager SI8000 (SONY) to trace column deflection, and the deflection distance was quantified using the video analysis software KINOVEA (Kinovea).

Force was estimated based on the previous reportand calculated using the following equation. Here, F (μN) is the estimated tension; E (kPa) is Young's modulus of the material; I is the cross-sectional secondary moment; L (mm) is the height of the displacement measurement point; x (mm) is the average height of the myocardial tissue attachment, and δ (μm) is the pillar displacement. In this system, E = 83, I = 0.00307, L = 0.5, and substituting x = 0.5 yields,

$F\ [\mu N] = 6.11\ [\mu N/\mu m] \times \delta\ [\mu m]$.

6. qPCR

For qPCR analysis, total RNA samples were reverse-transcribed using the QuantiTect Reverse Transcription Kit (Qiagen). Quantitative real-time PCR (qPCR) was performed using THUNDERBIRD SYBR Green qPCR Mix (TOYOBO). Relative expression levels of the target genes were normalized to the expression of an internal control gene (*RPS28*) using the comparative Ct method. The primers used for qPCR were as follows:

(Human)

RPS28, GGTCTTGGATGTCGGGTTC, AGGAGCATCTCAGTTACGTGTG;
CETN2, ACTCCTTTGGCTATGGCCTC, GCTCAGGCTTAGGGCTCATT;
XRCC4, AGCTTCAAGAAAAGGAAAAGCCT, TCTGGGCTGCTGTTTCTCAG;
SMC5, ATCTCGATGGAGAACTTCCTAACA, CTCGTCCCATGAAAGCAGGT;
MBD4, CTCAATCGGACCTCAGGCAA, GTCTGCGGTTCTTGCTACCT;
EXO1, ACTGTTTCAGATGCCCCACA, TCTGACAGCTCTGCACTTCTT;
ATF2, CATGTGAATTCTGCCAGGCAAT, CTCGTTGGTAAAACGCTGGC.

(Mouse)

Cetn2, CAGATGAGGAACTGCAGGAAATG, CGCAGGAACTCTTGCTCATTG
Xrcc4, TGGAGTACTGAGAGGTAGCCA, AAACTGTTGCAGTCCAGGCT

7. Immunostaining of iPSCMs

To immunostain iPSCMs, cells were fixed with 4% paraformaldehyde (Nacalai Tesque) for 10 min, permeabilized with 0.1% Triton X-100 (Wako) for 5 min, and blocked with PBS containing 5% goat serum (Wako) for 1 h at room temperature. The samples were incubated with primary antibodies overnight at 4° C, and Alexa Fluor-conjugated secondary antibodies for 1 h at room temperature. Nuclei were counter-stained with DAPI (Thermo Fisher). Images were captured for the downstream analysis with the following imaging devices: Operetta High-Content Imaging System (PerkinElmer) and a confocal microscope (Carl Zeiss, LSM 880). For γH2AX quantification, images were analyzed with a software (Harmony) accompanied with Operetta. First, nuclei were segmented using the DAPI channel. Next, cell boundaries were defined from the TNNT2 staining images, and cells in which the TNNT2 fluorescence intensity in the cytoplasm exceeded a certain threshold were defined as cardiomyocytes. γH2AX foci were quantified only within the nuclear mask of cardiomyocytes. Primary antibodies were lamin A/C (4C11) (Cell Signaling Technologies, #4777, 1:400), TNNT2 (13-11) (Thermo Fisher, #MA5-12960, 1:500), γH2AX (Abcam, ab2893, 1:400), and VDR (9A7) (Thermo Fisher, #MA1-710, 1:100).

8. Creation of *Lmna* p.R225X mutant mice

The Lmna p.R225X knock-in mice were generated using CRISPR-Cas9. The sgRNA was synthesized using the CUGA7 gRNA synthesis kit (NipponGene) following the manufacturer's instructions, and Cas9 was purchased from NipponGene. The sequence of the gRNA was as follows:

gRNA, CCAAGCGCCGGCATGAGACGcGG

The sequence of the ssODN was as follows:

CAAACTCTCGCTGCTTCCCGTTATCGATCTCCACAAGCtaCGTCTCATGCCGGCG CTTGGTCTCACGCAGTTCCTGAAA

Mouse zygotes were obtained by mating superovulated BDF1 females and WT BDF1 males. RNAs and ssODNs were mixed just before microinjection into the cytoplasm and pro-nuclei of zygotes, and the injected embryos were incubated at 37° C until they were transferred into pseudo-pregnant ICR females at the two-cell stage. All mice were purchased from the Sankyo Labo Service Corporation.

9. Creation of pressure-loaded mice, administration of the drug

Male C57BL/6J mice aged 7–8 weeks and weighing 20–25 g were purchased (CLEA Japan Inc.) and used for TAC experiments. All mice were housed in separate cages at a maximum of 6 mice per cage in a specifically-pathogen-free, temperature-controlled vivarium under a 12-h light/dark cycle with ad libitum access to food and water. Pressure overload was induced

by TAC as previously described11. The transverse aorta was constricted with a 6–0 silk suture paralleled with a 27-gauge blunt needle, which was removed after the constriction. Sham-operated mice that underwent a similar surgical procedure without aortic constriction were used as a control (sham) and were examined 2-, 4-, and 6-weeks following surgery. Paricalcitol (5 μg/kg, Selleck) or vehicle (water) was administered every other day. Paricalcitol or vehicle was administered by intraperitoneal for 4 weeks (8-12 wks after birth) to *Lmna* R225X miceday, and for 6 weeks to TAC mice from 2 days after TAC surgery. The administered dose was calculated according to body weight. Plasma calcium and inorganic phosphate were measured using enzymatic assays after two weeks administration. Transthoracic echocardiography was performed following a protocol described previously. Mice were anesthetized by 2% isoflurane inhalation during the echocardiographic examination to minimize aberrant data. Two-dimensional short-axis view and M-mode echocardiography were recorded with the Vevo2100 Ultrasound System (FUJIFILM Visual Sonics). Cardiac wall chamber dimensions and fractional shortening were determined with these imaging techniques. For echocardiographic measurements, each mouse was treated as one biological replicate. TAC operation and echocardiography evaluation were performed by the same blinded operator to reduce variability.

10. Staining of mouse tissue

For immunostaining, mouse hearts were fixed in 10% formalin, embedded in paraffin, and sectioned to a 5 μm thickness. The sections were incubated with anti-γH2AX antibody (Abcam, ab2893, 1:100) overnight at 4° C. A secondary antibody (Alexa Fluor 594 goat anti-rabbit antibody: Invitrogen, 1:100), fluorescein-conjugated wheat germ agglutinin (WGA; 1:200), and DAPI (Biotium) were also applied. Images were obtained using an LSM 880 META confocal microscope (Zeiss). For the assessment of nuclear morphology in mice, the images were analyzed using Image J (NIH).

11. Data-sharing Statement

The RNA-seq data generated are available through Gene Expression Omnibus (GEO) (accession number is GSE182219, https://www.ncbi.nlm.nih.gov/geo/query/acc.cgi?acc=GSE182219). The data sets generated or analyzed during this study are available from the corresponding author on reasonable request.

12. Statistical Analysis

All data analyses were performed using GraphPad Prism 10 (GraphPad Software Inc.).

All statistical tests were two-sided, and P values <0.05 were considered statistically significant. Data are presented as mean ± s.e.m. unless otherwise indicated. Experimental units and sample sizes are specified in the corresponding figure legends.

Comparisons between two independent groups were performed using two-sided unpaired Student's t-tests (Figures 1D, 1F, 2D, 2E, and 4G). Comparisons among three or more independent groups were performed using ordinary one-way ANOVA followed by Tukey's multiple-comparisons test (Figures 2A and 4E). For tissue γH2AX quantification, multiple nuclei were measured within each mouse. These data were analyzed using nested one-way ANOVA, with nuclei nested within mouse and mouse treated as the higher-level experimental unit (Figures 3C and 4C).

Longitudinal echocardiographic data (Figures 3D and 4D) were analyzed using a mixed-effects model for repeated measures fitted by restricted maximum likelihood. Group, time, and the group-by-time interaction were included as fixed effects, with time treated as a categorical variable. Mouse identity was included as a random intercept to account for repeated measurements within each mouse; no random slope for time was included. The model used a compound-symmetry covariance structure. Sphericity was not assumed, and the Geisser–Greenhouse correction was applied. Missing values were not imputed, and all available observations were included in the mixed-effects analysis.

13. Definition of replicates in iPSCM experiments

An independent cardiac differentiation batch was defined as a separate differentiation initiated from undifferentiated iPSCs on a different day. For γH2AX quantification and qPCR analysis, experiments were performed using two independent cardiac differentiation batches. For the quantification of γH2AX, multiple images (10 or more) were captured from three wells under each condition (control or *Lmna* R225X) within a single differentiation batch, and the nuclear measurements were pooled at the well level prior to statistical analysis. The graph was generated using a total of six wells from two differentiation batches, with each data point representing a single well. For qPCR, we prepared two DMSO-treated samples and three VD2-treated samples within a single differentiation batch and repeated the experiment using a separate differentiation batch. Before statistical analysis, we averaged the data from the technical replicates. The graphs were generated as biological replicates using data from each sample across the two differentiation batches. For microtissue force measurements, engineered cardiac microtissues were generated from one cardiac differentiation batch, and each data point represents one microtissue.

Supplementary Table 1. Plasma calcium and inorganic phosphate concentration after paricalcitol treatr

	**Vehicle/DMSO** (n = 6)	**Paricalcitol** (n = 6)	*P* value
Plasma calcium (mg/dL)	9.20 ± 0.20	9.68 ± 0.10	0.061
Plasma inorganic phosphate (mg/dL)	8.37 ± 0.41	9.10 ± 0.26	0.165

nent.
